# The 2025 AHA/ACC hypertension guidelines: implications for cardiovascular and renal risk in patients with diabetes

**DOI:** 10.1186/s40842-025-00239-3

**Published:** 2025-08-26

**Authors:** Gaetano Santulli

**Affiliations:** 1 Department of Medicine, Albert Einstein College of Medicine, 10461 New York City, NY USA; 2 Academic Research Unit, Department of Advanced Biomedical Sciences, International Translational Research and Medical Education (ITME) Consortium, “Federico II” University, Naples, 80131 Italy; 3 Department of Molecular Pharmacology, Wilf Family Cardiovascular Research Institute, Einstein Institute for Aging Research, Einstein-Mount Sinai Diabetes Research Center (ES-DRC), Einstein Institute for Neuroimmunology and Inflammation (INI), Fleischer Institute for Diabetes and Metabolism (FIDAM), Albert Einstein College of Medicine, 10461 New York City, NY USA

## Abstract

Hypertension remains a major contributor to cardiovascular and renal complications in patients with diabetes mellitus, increasing the risk of macrovascular and microvascular disease. The 2025 AHA/ACC hypertension guidelines maintain a diagnostic and treatment threshold of 130/80 mmHg, emphasizing earlier and more intensive blood pressure control to reduce cardiovascular events, stroke, heart failure, and progression of diabetic nephropathy. Evidence from clinical trials and meta-analyses supports the benefits of tighter blood pressure targets, while acknowledging potential risks such as hypotension, electrolyte disturbances, and acute kidney injury. Management strategies combine pharmacologic therapy with lifestyle interventions including dietary modification, physical activity, weight management, and smoking cessation. Individualized blood pressure targets are recommended for older or frail patients to balance safety and benefit. Home and ambulatory blood pressure monitoring are highlighted for detecting masked or nocturnal hypertension, enhancing risk stratification, and supporting treatment titration. The guidelines also emphasize integrated risk assessment and multidisciplinary management. The 2025 AHA/ACC hypertension guidelines provide an evidence-based, patient-centered framework to optimize cardiovascular and renal outcomes in patients with diabetes, promoting early intervention, individualized therapy, and comprehensive risk reduction.

## Introduction

Hypertension remains a primary driver of morbidity and mortality in patients with diabetes mellitus, with an established role in accelerating macrovascular and microvascular complications [1]. Cardiovascular disease (CVD) and chronic kidney disease (CKD) are particularly prevalent among individuals with type 2 diabetes, with elevated blood pressure (BP) further compounding risk [2]. The recently released 2025 American Heart Association/American College of Cardiology (AHA/ACC) hypertension guidelines have introduced significant updates that refine BP thresholds, treatment targets, and therapeutic strategies [3]. These revisions aim to optimize cardiovascular risk reduction while balancing safety and patient-centered care. This commentary discusses the relevance and implications of the 2025 AHA/ACC guidelines specifically for patients with diabetes, contrasting them with prior guidelines and current European recommendations, and highlights the clinical, mechanistic, and public health considerations underlying these recommendations.

## Rationale for lower blood pressure targets in diabetes

The 2025 guidelines maintain the 130/80 mmHg cutoff for both diagnosis and initiation of treatment [3], reaffirming the 2017 AHA/ACC approach [4]. This decision is supported by evidence from randomized clinical trials and meta-analyses indicating that patients with diabetes benefit from earlier and more intensive BP management. Landmark studies such as SPRINT (although diabetics were excluded) and ACCORD-BP, along with *post-hoc* analyses from ADVANCE and UKPDS, demonstrate that reductions in systolic blood pressure to below 130 mmHg are associated with meaningful reductions in cardiovascular events and progression of microvascular disease (Table 1).

**Table 1:** Side-by-side comparison table of the 130/80 mmhg cutoff vs. 140/90 mmhg cutoff for defining hypertension

	130/80 mmHg Cutoff	140/90 mmHg Cutoff
**Primary Rationale**	Aligns with evidence (e.g., SPRINT) showing benefits of earlier intervention and tighter BP control in high-risk groups	Historically based on epidemiologic and trial data linking ≥ 140/90 mmHg to increased risk; avoids overdiagnosis
**Population Labeled Hypertensive**	Significantly more (about 46% of U.S. adults)	Fewer (about 32% of U.S. adults)
**Impact on Early Detection**	Detects more patients with elevated cardiovascular risk earlier	May miss some at-risk individuals with BP 130–139/80–89 mmHg
**Effect on Cardiovascular Outcomes**	Potential for greater long-term risk reduction, especially in high-risk populations	Proven benefit in reducing CVD events at ≥ 140/90, but may delay intervention for moderate elevations
**Treatment Threshold**	Lifestyle changes recommended for all; medication typically reserved for high-risk patients or those with CVD, CKD, or diabetes	Lifestyle modification first; medication typically starts at ≥ 140/90 (or ≥ 130/80 in certain high-risk groups)
**Risk of Overtreatment**	Higher—more individuals may receive medication despite modest absolute risk	Lower—focuses treatment on those with more clearly elevated BP and higher risk
**Side Effects & Safety Concerns**	Greater potential for medication-related adverse effects (hypotension, falls, electrolyte disturbances) in low-risk individuals	Lower risk of overtreatment side effects
**Healthcare System Burden**	Increased—more frequent monitoring, counseling, and follow-up required	More limited patient load for monitoring and treatment
**Patient Psychological Impact**	More individuals labeled as hypertensive, possibly increasing anxiety or altering self-perception of health	Fewer individuals diagnosed; less anxiety but risk of delayed intervention
**Global Guideline Alignment**	Matches current U.S. AHA/ACC approach; differs from most European guidelines (still at 140/90)	Matches ESC and other regions’ recommendations

Elevated BP in diabetes exacerbates endothelial dysfunction, increases oxidative stress, and promotes inflammation, accelerating atherosclerosis. These pathophysiologic mechanisms justify early intervention, even for patients with mild to moderate elevations in BP. By lowering the diagnostic threshold, the guidelines enable clinicians to identify at-risk individuals earlier, potentially preventing the onset of target organ damage, including nephropathy, retinopathy, and left ventricular hypertrophy.

## Evidence supporting the 130/80 mmhg threshold

Meta-analyses examining the effect of intensive versus standard BP control in diabetic populations have consistently demonstrated reductions in stroke, myocardial infarction, and heart failure events with tighter BP targets. In ACCORD-BP, targeting a systolic BP < 120 mmHg resulted in a significant reduction in stroke risk, though the overall composite cardiovascular endpoint was not statistically significant. However, the trial highlighted the potential trade-offs of intensive therapy, including increased incidence of hypotension, syncope, and electrolyte disturbances.

The 2025 AHA/ACC guidelines emphasize a risk-based approach, recommending pharmacologic therapy for patients with diabetes who exceed 130/80 mmHg and possess additional cardiovascular risk factors or evidence of target organ damage. Lifestyle modification remains foundational, and pharmacologic therapy is introduced when lifestyle measures alone are insufficient to achieve targets (Table 1).

## Treatment strategies and drug selection

For patients with diabetes, the guidelines reinforce the preferential use of renin-angiotensin system inhibitors—ACE inhibitors or ARBs—particularly in the presence of albuminuria or CKD, due to their proven nephroprotective effects. Thiazide-like diuretics and calcium channel blockers are recommended as adjuncts when monotherapy fails to achieve targets, while beta-blockers are reserved for specific indications such as coronary artery disease or heart failure with reduced ejection fraction. The guidelines also emphasize medication adherence, simplification of regimens, and consideration of patient comorbidities, age, and frailty.

## Individualized blood pressure targets

A key advancement in the 2025 recommendations is the focus on individualized BP targets. While < 130/80 mmHg is generally recommended for most adults with diabetes, the guidelines acknowledge circumstances in which less stringent targets may be appropriate. Older adults with frailty, autonomic dysfunction, or polypharmacy are at increased risk of hypotension, falls, and medication-related adverse events [5]. In these populations, targets might be relaxed, emphasizing safety while still striving for meaningful risk reduction.

The individualized approach also accounts for cardiovascular risk stratification. Patients with prior myocardial infarction, stroke, or heart failure may benefit from more aggressive targets, whereas those with lower baseline risk may prioritize lifestyle modification with careful monitoring before initiating pharmacotherapy.

## Integration with lifestyle modification

The 2025 guidelines strongly advocate lifestyle interventions as the cornerstone of hypertension management in diabetes. Dietary approaches, including sodium restriction, adherence to DASH-like diets modified for glycemic control, weight management, and regular physical activity, are emphasized. For patients with type 2 diabetes, caloric restriction and weight loss have synergistic effects on BP reduction and glycemic control, further mitigating cardiovascular risk. Smoking cessation, moderation of alcohol intake, and sleep optimization are also recommended.

## Home and ambulatory blood pressure monitoring

The guidelines underscore the utility of home BP monitoring and ambulatory BP measurement in patients with diabetes. These modalities help identify white-coat and masked hypertension, which are particularly prevalent in this population. Masked hypertension is associated with higher cardiovascular risk and may contribute to progression of diabetic nephropathy [6]. Nighttime BP assessment through ABPM provides additional prognostic information, as nocturnal hypertension is strongly linked to adverse renal and cardiovascular outcomes. Home monitoring enhances patient engagement, enables dose titration, and supports adherence.

## Potential advantages of the 2025 approach in diabetes

1. Earlier Detection and Intervention: By lowering the diagnostic threshold, more patients with elevated cardiovascular risk are identified and treated before significant target organ damage occurs.
2. Enhanced Risk Reduction: Tighter BP control has been linked to reduced rates of stroke, myocardial infarction, heart failure, and progression of diabetic nephropathy.
3. Integration with Multimodal Risk Management: BP control is emphasized alongside lipid and glycemic management, reflecting the multifactorial nature of cardiovascular risk in diabetes.
4. Patient-Centered Care: Individualized targets allow clinicians to balance benefits and risks, improving safety and adherence.

## Potential disadvantages and considerations

1. Risk of Overtreatment: Expanding the hypertensive population increases the number of patients receiving pharmacologic therapy, potentially exposing low-risk individuals to adverse effects.
2. Resource Intensiveness: More frequent monitoring, ABPM utilization, and follow-up visits may increase the healthcare burden.
3. Medication Side Effects: Intensive therapy can precipitate hypotension, electrolyte disturbances, acute kidney injury, and orthostatic symptoms, particularly in older adults or those with autonomic neuropathy.
4. Psychological Impact: More patients may be labeled as hypertensive, which could increase anxiety or affect perceived health status.

## Comparison with European guidelines

The 2025 AHA/ACC recommendations differ from the 2024 ESC guidelines [7], which generally maintain a 140/90 mmHg threshold for diagnosis and initiation of therapy, though they recognize 130–139/80–89 mmHg as elevated risk requiring lifestyle intervention [7]. European targets for pharmacologic therapy in diabetics are individualized but often less aggressive than AHA/ACC recommendations, reflecting a more conservative approach aimed at minimizing overtreatment and adverse effects (Table 2). Despite these differences, both guidelines emphasize the importance of ACE inhibitors/ARBs in patients with diabetes and albuminuria, lifestyle modification, and comprehensive cardiovascular risk reduction [8].

**Table 2:** Comparison of AHA 2017, AHA 2025, and ESC 2024 hypertension guidelines, with a focus on patients with diabetes

	AHA/ACC 2017	AHA/ACC 2025	ESC 2024
**Hypertension definition**	≥ 130/80 mmHg	≥ 130/80 mmHg	≥ 140/90 mmHg
**Elevated BP / prehypertension**	120–129/<80 mmHg	120–129/<80 mmHg	130–139/85–89 mmHg (lifestyle intervention only)
**BP treatment initiation for diabetes**	≥ 130/80 mmHg with cardiovascular risk or organ damage	≥ 130/80 mmHg with cardiovascular risk or organ damage	≥ 140/90 mmHg; 130–139/80–89 mmHg if high CV risk (lifestyle first, meds if insufficient)
**BP target in treated diabetics**	< 130/80 mmHg	< 130/80 mmHg (individualized in frail/older adults)	130–139/70–79 mmHg (lower systolic for younger/high-risk, relax in older/frail)
**Preferred first-line agents**	ACEi or ARB (especially with albuminuria)	ACEi or ARB (with albuminuria/CKD); Thiazide-like diuretics and CCBs as add-on	ACEi or ARB; CCB or diuretics as add-on; combination therapy encouraged
**Intensive therapy evidence**	ACCORD-BP, SPRINT (non-diabetic included)	ACCORD-BP, meta-analyses support intensive control (< 130 mmHg)	ACCORD-BP, meta-analyses; recommend less aggressive in elderly/frail
**Lifestyle modification emphasis**	DASH diet, sodium restriction, weight loss, physical activity	DASH-like diet, sodium restriction, weight loss, exercise, alcohol moderation, smoking cessation	Similar: DASH, sodium restriction, weight management, exercise, alcohol moderation, smoking cessation
**Home/ambulatory BP monitoring**	Recommended for white-coat/masked hypertension	Strongly recommended, including ABPM for nighttime BP	Recommended, especially for masked/white-coat hypertension
**Hypotension / adverse events caution**	Individualize in elderly/frail	Individualized targets to avoid hypotension, falls, AKI	Strong emphasis on safety in older adults, frail, CKD, autonomic neuropathy
**Integration with CV risk management**	Lifestyle + pharmacologic therapy integrated with glycemic and lipid control	Strong emphasis on multimodal risk reduction (BP, lipids, glucose, lifestyle)	Similar, risk-based approach, slightly more conservative BP targets

Key takeaways:

• Both AHA 2017 and 2025 use 130/80 mmHg as diagnostic and treatment thresholds, whereas ESC 2024 retains a higher threshold for initiation (140/90 mmHg)

• AHA 2025 reinforces individualized therapy, particularly in older or frail patients

• All guidelines emphasize ACEi/ARB in diabetes, lifestyle interventions, and use of combination therapy if targets are not achieved

• Home and ABPM monitoring are highlighted in 2025 more strongly than in 2017, reflecting an emphasis on precise BP control and identification of masked hypertension

• ESC guidelines remain more conservative, prioritizing safety in the elderly while recognizing the value of earlier intervention in high-risk patients

## Major clinical implications

For practicing clinicians, the 2025 AHA/ACC guidelines emphasize a multifaceted, proactive approach in managing hypertension among patients with diabetes. Key clinical implications include:

- Earlier engagement with pharmacologic therapy for patients with elevated BP and high cardiovascular risk.
- Frequent and accurate BP monitoring, including home measurements and ABPM when indicated.
- Integration of antihypertensive therapy with glycemic and lipid management, optimizing overall cardiovascular protection.
- Patient education and empowerment, highlighting lifestyle modification, adherence, and self-monitoring.
- Judicious use of individualized targets in older or frail patients to avoid adverse events.

Implementation of these guidelines has the potential to significantly reduce cardiovascular morbidity and mortality in patients with diabetes, but must be balanced with safety and patient-centered considerations.

## Cardiovascular-kidney-metabolic (CKM) syndrome

The 2025 AHA/ACC Hypertension Guidelines address the management of Cardiovascular-Kidney-Metabolic (CKM) syndrome, a condition characterized by the interplay of cardiovascular disease, chronic kidney disease, and metabolic disorders, which has seen increased incidence following the COVID-19 pandemic [9]. The guidelines emphasize early identification of elevated blood pressure and associated risk factors, incorporating integrated risk assessment tools to evaluate combined cardiovascular and renal risk. Management strategies prioritize lifestyle interventions, including dietary modifications, regular physical activity, weight control, and smoking cessation, alongside pharmacologic therapies such as ACE inhibitors, ARBs, and SGLT2 inhibitors for patients with diabetes or CKD. A multidisciplinary, team-based approach is recommended to ensure coordinated care across cardiology, nephrology, endocrinology, and primary care, aiming to mitigate the progression of CKM syndrome and improve long-term outcomes in this high-risk population [10].

## Future directions

While the 2025 guidelines provide a comprehensive framework, several areas warrant further research:

- Long-term outcomes of intensive BP lowering in diverse diabetic populations, including those with type 1 diabetes, older adults, and patients with multimorbidity.
- Optimization of combination therapy, balancing efficacy, adherence, and safety.
- Integration of novel monitoring technologies, such as continuous wearable BP devices, to enhance risk stratification and treatment titration.
- Cost-effectiveness analyses, assessing the impact of expanded diagnosis and earlier intervention on healthcare systems.
- Exploration of patient-reported outcomes, including quality of life, adherence, and psychosocial impact, in relation to lower BP targets.

## Conclusions

The 2025 AHA/ACC hypertension guidelines represent a paradigm shift in the management of blood pressure for patients with diabetes, emphasizing earlier detection, tighter targets, and individualized therapy. By lowering the threshold to 130/80 mmHg, the guidelines aim to reduce cardiovascular and renal complications and to integrate hypertension management into a broader, risk-based strategy. While these recommendations offer the potential for significant improvements in outcomes, careful consideration of adverse effects, patient characteristics, and healthcare resources remains essential. The guidelines underscore the importance of combining pharmacologic therapy with lifestyle interventions, rigorous monitoring, and patient-centered care to optimize the management of hypertension in diabetes. Clinicians must balance the benefits of intensive therapy with potential risks, tailoring treatment to individual patient profiles and comorbidities. Overall, the 2025 recommendations provide a robust evidence-based framework for improving cardiovascular and renal outcomes in this high-risk population, and represent a critical update for clinicians managing patients with diabetes worldwide.
